# ACTIV trials: Lessons learned in trial design in the setting of an emergent pandemic

**DOI:** 10.1017/cts.2024.1

**Published:** 2024-10-15

**Authors:** Maryam Keshtkar-Jahromi, Kevin J. Anstrom, Christina Barkauskas, Samuel M. Brown, Eric S. Daar, William Fischer, Kevin W. Gibbs, Elizabeth S. Higgs, Michael D. Hughes, Prasanna Jagannathan, Lisa LaVange, Christopher J. Lindsell, Seema U. Nayak, Roger Paredes, Mahesh Parmar, Ithan D. Peltan, Michael Proschan, Matthew S. Shotwell, David M. Vock, Tammy Yokum, Stacey J. Adam

**Affiliations:** 1 Division of Microbiology and Infectious Diseases, National Institute of Allergy and Infectious Diseases, National Institutes of Health, Rockville, MD, USA; 2 Gillings School of Global Public Health, University of North Carolina at Chapel Hill, Chapel Hill, NC, USA; 3 Duke Clinical Research Institute, Duke University, Durham, NC, USA; 4 Pulmonary and Critical Care Medicine, Intermountain, Salt Lake City, UT, USA; 5 Division of HIV Medicine, Lundquist Institute at Harbor- University of California Los Angeles Medical Center, Los Angeles, CA, USA; 6 Division of Pulmonary Diseases and Critical Care Medicine, University of North Carolina at Chapel Hill, Salt Lake City, UT, USA; 7 Wake Forest University School of Medicine, Winston-Salem, NC, USA; 8 Division of Clinical Research, National Institute of Allergy and Infectious Diseases, National Institutes of Health, Bethesda, MD, USA; 9 Department of Biostatistics, Harvard TH Chan School of Public Health, Boston, MA, USA; 10 Stanford University, Stanford, CA, USA; 11 Department of Biostatistics, University of North Carolina at Chapel Hill, Chapel Hill, NC, USA; 12 Department of Biostatistics and Bioinformatics, Duke University, Durham, NC, USA; 13 Division of Microbiology and Infectious Diseases, National Institute of Allergy and Infectious Diseases, National Institutes of Health, Rockville, MD, USA; 14 Department of Infectious Diseases, Hospital Germans Trias and irsiCaixa, Badalona, Spain; 15 Medical Research Council Clinical Trials Unit, University College of London, London, WC, UK; 16 Department of Pulmonary and Critical Care Medicine, Intermountain, Salt Lake City, UT, USA; 17 National Institute of Allergy and Infectious Diseases, National Institutes of Health, Rockville, MD, USA; 18 Vanderbilt University, Nashville, USA; 19 Division of Biostatistics & Health Data Science, School of Public Health, University of Minnesota, Minneapolis, MN, USA; 20 Foundation for the National Institutes of Health, North Bethesda, MD, USA

**Keywords:** ACTIV, COVID-19, clinical trial, design, lessons

## Abstract

Accelerating COVID-19 Treatment Interventions and Vaccines (ACTIV) was initiated by the US government to rapidly develop and test vaccines and therapeutics against COVID-19 in 2020. The ACTIV Therapeutics-Clinical Working Group selected ACTIV trial teams and clinical networks to expeditiously develop and launch master protocols based on therapeutic targets and patient populations. The suite of clinical trials was designed to collectively inform therapeutic care for COVID-19 outpatient, inpatient, and intensive care populations globally. In this report, we highlight challenges, strategies, and solutions around clinical protocol development and regulatory approval to document our experience and propose plans for future similar healthcare emergencies.

## Introduction

**Figure 1.**
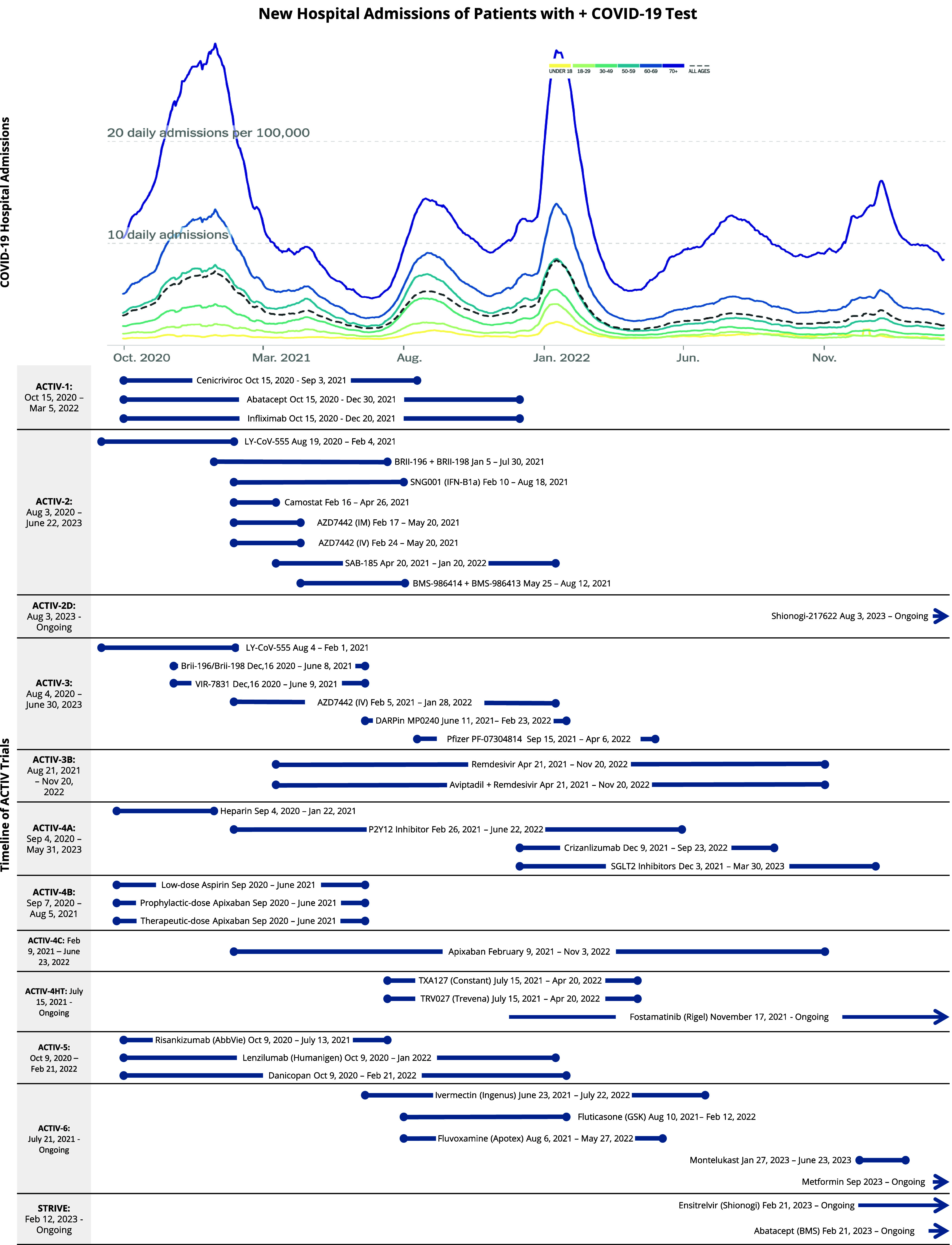
Timeline of new hospital admissions in the US and enrollment dates of agents within ACTIV. Permissions received to use the hospitalization chart showing the number of individuals per 100,000 that were newly admitted to a hospital with COVID-19 each day sourced from the “New York Times” (https://github.com/nytimes/covid-19-data). The ACTIV trials enrolled patients to test the above agents. Each agent’s timeline is shown from the launch of that study arm through to the date that the last patient was enrolled in the study. The ACTIV timeline on the left shows the date of the launch of the overall master protocol through patient follow-up times after the agent arms completed enrollment. A few agents being studied in the suite of ACTIV trials continue to enroll (with over 26,000 patients) at the time of this publication, as indicated by the ongoing arrows. ACTIV = Accelerating COVID-19 Therapeutic Interventions and Vaccines; US = United States of America.

On April 17, 2020, the United States Government (USG) initiated the National Institutes of Health (NIH) led public-private partnership known as Accelerating COVID-19 Treatment Interventions and Vaccines (ACTIV). The overall goal of ACTIV was to rapidly develop SARS-CoV-2 medical countermeasures to mitigate COVID morbidity and mortality and accelerate the end of the pandemic by leveraging and synergizing USG and private industry research capabilities [1]. At the initiation of ACTIV, the United States (US) was experiencing over 2,000 daily COVID deaths and 23,000 new cases per day (Fig. 1). Little was known about the pathogenesis of SARS-CoV-2, and results from the randomized controlled trial of remdesivir within the Adaptive COVID-19 Treatment Trial (ACTT-1) [2,3] and dexamethasone within the Randomized Evaluation of COVID-19 Therapy (RECOVERY) trial [4] were not yet known. What was being observed was rapid progression to acute respiratory distress syndrome (ARDS) in some patients, identification of comorbidities possibly associated with high risk of progression, evidence of thrombosis in patients with active disease, early autopsy data showing *in situ* thrombosis in the lungs, and relative paucity of illness in young children.

With limited understanding of disease pathogenesis, the ACTIV Therapeutics-Clinical Working Group (Tx-Clin WG) was tasked with developing a systematic process for identifying promising treatment candidates and for rapidly launching rigorous regulatory-level trials to evaluate therapeutic candidates [5]. The ACTIV Tx-Clin WG chose to establish master protocols within which agents would be tested. Master protocols, simply put, allow multiple agents to be investigated under a single protocol. While master protocols can be complex to develop and launch, they allow for efficiency gains in design and operations when testing multiple agents in a similar context. The decision to use master protocols has been previously discussed in detail [5].

At the outset, the ACTIV Tx-Clin WG decided upon three master protocols, based on population and therapeutics targets: therapeutics for immune modulation in inpatients (ACTIV-1), antivirals in outpatients (ACTIV-2), and antivirals in inpatients (ACTIV-3). Shortly thereafter, a set of three master protocols were designed to test anticoagulation strategies (ACTIV-4A, B, and C) in inpatients, outpatients, and convalescent patients, respectively. Somewhat later, ACTIV added four additional master protocols: ACTIV-3B for COVID ARDS patients, the Big Effect Trial (ACTIV-5) to evaluate candidate therapeutics for an early large mortality benefit that could then be verified in ACTIV-1 or ACTIV-3, ACTIV-4HT to test host-targeted agents, and finally, a decentralized outpatient master protocol for repurposed agents (ACTIV-6). The final master protocol was a new addition to the ACTIV-2 protocol (ACTIV-2B/C and D), with only ACTIV-2D launching. The agents selected for the trials are detailed in Figure 1 according to their start and end times relative to the pandemic.

**Figure 2.**
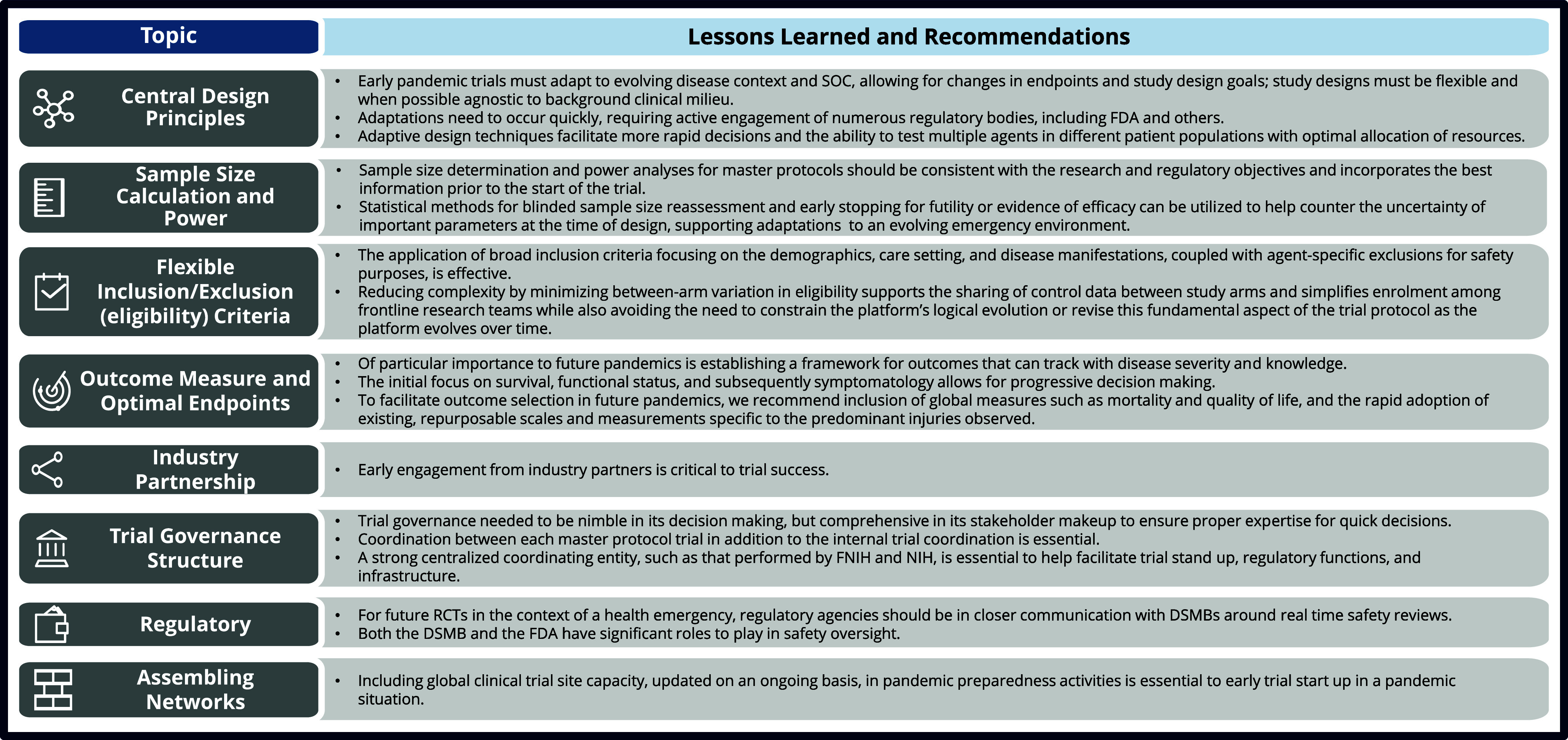
Summary of lessons learned for master protocol design for emergency situations. The high-level takeaway lessons learned from the design of the ACTIV master protocols and potential solutions that may be utilized in future pandemics. DSMB = Data Safety Monitoring Board; FDA = US Food and Drug Administration; FNIH = Foundation for the National Institutes of Health; NIH = National Institute of Health; RCT = randomized controlled trial; SOC = standard of care.

To date, the ACTIV protocols have enrolled over 26,000 patients and evaluated 37 agents, with progress summarized in the ACTIV Overview and Tx-Clin WG reports in this issue. When developing these trials, ACTIV learned much about the specifics of designing master protocols, as well as many general clinical trial design issues that need to be managed in the setting of a pandemic. This paper focuses on learnings that will likely have implications for the research response to future health emergencies. For each challenge area discussed, lessons learned are provided and summarized in Figure 2.

### Protocol design in the context of the unknown

As we now know, SARS-CoV-2 infection results in a broad spectrum of clinical disease, ranging from asymptomatic carriage to mild respiratory illness and, in its most severe forms, hospitalization with fulminant COVID-19 respiratory failure. However, in the early days of this novel disease, these diverse clinical manifestations coupled with limited knowledge of the natural history and pathogenesis of COVID-19 posed distinct and unprecedented challenges for the ACTIV master protocols. The dearth of understanding affected multiple aspects of trial design including: (1) type and timing of outcome measures, (2) patient eligibility, and (3) selection of candidate therapies. A specific challenge for master protocols is that as the scientific understanding improves, many aspects of the trial design cannot be feasibly changed within a master protocol. As the pandemic evolved and the anticipated number of agents to be studied became clearer and more modest, early hospitalization futility assessments were removed from the ACTIV-4 and ACTIV-5 trials, but not from the ACTIV-3 trial. While time-to-recovery outcomes and organ failure-free outcomes were available and could be adapted from existing clinical trial experience, the use of cross-sectional assessments of outcomes measured at a specific study day was common for convenience and ease of interpretation. Even when using ordinal scale outcomes, with incomplete knowledge of COVID convalescence selecting a single study day for outcome ascertainment runs the risk of missing treatment effects if the chosen day is too early (before a difference among treatment groups can be observed) or too late (after nearly everyone has improved).

As with outcomes, limited understanding and emerging knowledge of viral-host interactions influenced eligibility criteria in the ACTIV platforms. Selecting the appropriate patient population and balancing disease severity and patient safety at enrollment proved challenging. Concern for potentiation of inflammation resulted in exclusion of critically ill patients from some ACTIV platforms, like ACTIV-3, and not others, like ACTIV-1, with the choice depending on the mechanism of action of agents being studied. Likewise, use of advanced oxygen delivery devices, such as heated humidified high-flow nasal oxygen, in step-down units, resulted in a substantial number of patients with COVID ARDS being treated outside of an ICU and not being enrolled. For example, because of the monitoring required for execution of the ACTIV-3B protocol, many sites did not include patients with ARDS who were being managed on a step-down unit. While eligibility challenges are important for all trials, given the scope of master protocols with multiple often unknown therapies planned for study, it is critical to apply the broadest criteria possible and then impose restrictions based on individual agents as they are added to the platform.

Emerging knowledge of the SARS-CoV-2 pathogen and pathogenesis, although still incomplete, directly informed platform design and candidate therapy selection. Early reports surrounding hypercoagulability resulted in establishing the ACTIV-4A, -4B, and -4C platforms to evaluate antithrombotic therapies. Likewise, recognition of angiotensin-converting enzyme 2 (ACE2)-SARS-CoV-2 binding as a key pathway for viral entry into host cells led the ACTIV-4HT platform to evaluate renin-angiotensin-system modulating agents. The emergence of effective therapies, new SARS-CoV-2 variants, changes in care practices, and decreases in mortality rates over time all influenced protocol design, trial power, and validity of outcomes. Despite the challenge of limited and emerging knowledge, by establishing a continuum of master protocols rooted in a strategic learning-to-approval framework, ACTIV created an adaptable roadmap for accelerating medical interventions in future pandemics.

### ACTIV master protocol central design principles

Overall, eleven master protocols were established within the six platforms (Table 1). Each master protocol targeted a patient care setting (inpatient or outpatient), host response or virus, and disease manifestation. The master protocols typically evaluated therapeutic agents with respect to disease progression, time to recovery, mortality, and hospital resource utilization; trials of antithrombotic agents had prevention outcomes. The majority of the master protocols described a randomized, blinded, placebo-controlled platform study that allowed investigational drugs to be added and dropped during the study. ACTIV-4A opted for a more open-label, pragmatic design to facilitate a more formal comparison of the data from platform trials outside of the ACTIV initiative.

**Table 1.** Summary of design attributes for each ACTIV master protocol

Population	Master protocol	Phase	Drug class	Networks	Target sample size (per arm)	Agents tested	Trial contributions
Inpatient Studies	ACTIV-1	III	Host-targeted Immune Modulators	BARDA/NCATS + CTSA Program/TIN + CRO (TRI/DCRI/Syneos)	540	*Abatacept (BMS), Cenicriviroc (AbbVie), Infliximab (Janssen)*	Infliximab and Abatacept decrease mortality in hospitalized patients
	ACTIV-3	III	mAbs and Antivirals	NIAID INSIGHT + NHLBI PETAL + CTSN + VA	500-750	*mAbs (Lilly, Brii, GSK-Vir, AZ), DARPin (Molecular Partners), ritonavir/nirmatrelvir (Pfizer)*	Proved baseline SARS-CoV-2 NC antigen from the periphery and SARS-CoV-2 seropositivity status is predictive of progression of disease
	ACTIV-3B	III	Host-targeted Immune Modulators for ARDS	NIAID INSIGHT + NHLBI PETAL + CTSN + VA	620	*Aviptadil, VIP (NeuroRx)*	A master protocol to study patients with ARDS that can be adapted to future trials quickly
	ACTIV-4A	III	Host-tissue Directed Antithrombotics Vascular Integrity/ Thromboinflamm ation	NHLBI CONNECTS	1000	*LMWH, UFH, P2Y12 Inhibitors (Anti-platelet Agents), Crizanlizumab (Novartis), SGLT2 inhibitors*	Trials in anticoagulation identified heterogeneity of treatment effect, with a benefit to therapeutic anticoagulation in moderately ill hospitalized patients, and no benefit (with a signal towards harm) in the critically ill. This finding changed clinical guidelines and practice No benefit was identified for organ support-free days with the use of P2Y12 inhibitors or Crizanlizumab or SGLT2i
	ACTIV- 4HT	II/III	Host-tissue Targeted Therapies	NHLBI CONNECTS	600	*TXA127 (Constant), TRV027 (Trevena), Fostamatinib (Rigel)*	Provided robust evidence that modulation of the renin-angiotensin system with either angiotensin receptor blockade or MAS receptor activation does not improve outcomes for patients with severe Covid- 19
	ACTIV-5	II	Screen Promising Immune Modulators	NIAID + CRO	200 (expansion–500)	*Risankizumab (AbbVie), Lenzilumab (Humanigen), Danicopan (AZ)*	Demonstrated that smaller proof of concept studies can help identify if agents are likely to be successful prior to funding large pivotal trials
	STRIVE (severe ARI)	III	All agents relevant to acute viral respiratory diseases	NIAID INSIGHT	750	*Ensitrelvir (Shionogi)*	Formed in 2022 as a network of networks (ACTIV-1, 3, and 5), it is built to optimize a durable model that is agile and able to respond to infectious emergencies, as well as further innovate trial design including appropriate primary endpoint
Outpatient Studies	ACTIV- 2/2D	II/III	Antibody based therapies and Oral and inhaled antivirals	NIAID ACTG + CRO	Ph II = 110 Ph III = 600– 1000	*mAbs (Lilly, Brii Bio, RU-BMS, AZ), IFN-beta (Synairgen), camostat (Sagent), nPAB (SAB), Ensitrelvir (Shionogi)*	Lilly included data from the ACTIV-2 study in FDA EUA submission package AZ included data from ACTIV-2 study in FDA EUA submission package Brii-196/Brii-198 granted EUA in China
	ACTIV-4B	III	Host-tissue Directed Antithrombotics	NHLBI CONNECTS	1750	*Low-dose Aspirin, Prophylactic-dose Apixaban, Therapeutic-dose Apixaban (BMS)*	Determined that stable ambulatory outpatients newly diagnosed with COVID- 19 did not benefit from antithrombotic treatment as the thrombotic event rates in this population are low
	ACTIV-6	III	Existing Prescription and OTC Medications	NCATS CTSA and Trial Innovation Network + PCORnet + Conduct Clinical Trials + SignalPath	1200	*Ivermectin (Ingenus), fluvoxamine (Apotex), fluticasone (GSK), Montelukast (Accord), Metformin*	Novel methods and best practices for conducting a fully remote trial during a public health emergency Development of a novel composite outcome based on distribution of events related to time to recovery from symptoms, healthcare encounters (hospitalization, urgent care, emergency department) and death Trial design that was recognized as accurate and unbiased as well as patient- centered Definitive results on ivermectin and fluticasone that have been used to inform recommendations from COVID-19 Treatment Guidelines Committees
	ACTIV-4C (convale scent)	III	Host-tissue Directed Antithrombotics	NHLBI CONNECTS Network	2660	*Apixaban (BMS)*	Integrated an in-person recruiting and enrollment process with all study follow- up procedures conducted remotely by a centralized call service Addressed issues related to co-enrollment of participants into different randomized clinical trials Documented importance of incorporating quality-of-life assessment with clinical outcomes in a disease with a chronic component

ACTG = AIDS Clinical Trials Group; ACTIV = Accelerating COVID-19 Therapeutic Interventions and Vaccines; ARDS = Acute Respiratory Distress Syndrome; ARI = Acute Respiratory Infection; AZ = AstraZeneca; BMS = Bristol Myers Squibb; CONNECTS = Collaborating Network of Networks for Evaluating COVID-19 and Therapeutic Strategies; CTSN = Cardiothoracic Surgical Trials Network; CRO = Contract Research Organization; DCRI = Department of Clinical Research Informatics; EUA = Emergency Use Authorization; FDA = US Food and Drug Administration; INSIGHT = International Network for Strategic Initiatives in Global HIV Trials ; Lilly = Eli Lilly and Company; mAbs = Monoclonal Antibodies; NCATS = National Center for Advancing Translational Sciences; NHLBI = National Heart; Lung; and Blood Institute; NIAID = National Institute of Allergy and Infectious Diseases; OTC = Over-the-Counter; PETAL = Prevention and Early Treatment of Lung Injury; PCORnet = National Patient-Centered Clinical Research Network; SGLT2 = Sodium-glucose cotransporter-2; STRIVE = Strategies and Treatments for Respiratory Infections and Viral Emergencies; TIN = Trial Innovation Network; TRI = Technical Resources International, Inc; VA = Veterans Affairs.

ACTIV trial summaries with their impact on patients’ management and outcome are detailed in the final column of the table. Abbreviations within the table are detailed here.

Over time, protocols were expected to adapt to incorporate emerging standards of care (SOC); each agent was intended to be evaluated as add-on therapy to the prevailing SOC. Indeed, the master protocols were explicitly designed to learn fast and adapt to change. This was demonstrated by ACTIV-2 and -3 where there was limited clinical safety and efficacy data for novel products to support a phase 3 study. As a result, the master protocols were designed as placebo-controlled phase 2/3 studies with pre-specified graduation rules based on intermediate markers for efficacy. During phase 2, the Data Safety Monitoring Board (DSMB) assessed in real time whether graduation criteria were met. If so, the agent transitioned to a phase 3 clinical endpoint trial that efficiently included all participants in the final sample for the phase 3 analysis.

For the ACTIV trials, development and implementation of a master protocol for a placebo-controlled trial facilitated the ability to adapt by discontinuing less-promising agents and adding new agents of interest, increased efficiency through sharing of control patients among study arms, and avoided duplication of effort in terms of infrastructure, trial governance, information systems (EDC [electronic data capture], web-based randomization, etc.), and other study management aspects.

The need to respond to a rapidly evolving pandemic made the use of adaptive designs attractive for ACTIV master protocols. Some protocols incorporated Bayesian decision rules for agents to transition from one stage of the study to another (similar to a phase 2/3 adaptive design); some incorporated Bayesian monitoring for early stopping decisions based on the agent probability of success; others utilized more standard frequentist methods like blinded sample-size reassessment and alpha and beta spending functions to assess the evidence for early stopping for efficacy or lack of efficacy. Use of adaptive design techniques facilitated more rapid decisions in each platform trial and the ability to test multiple agents in different patient populations (disease severity) with optimal allocation of resources (e.g., unpromising agents were discontinued early to make room for others). Additional details about the variety of adaptive designs used across the master protocols can be found in the ACTIV statistical lessons learned manuscript in this journal issue.

### Sample-size calculation and power

Sample-size determination and power and control of pairwise error were key considerations in designing the ACTIV master protocols. From the beginning, the goal was to ensure each protocol could produce evidence sufficient for the US Food and Drug Administration (FDA) to support emergency use authorization (EUA) for each agent, and power needed to be sufficient to detect moderate, clinically meaningful treatment effects. An adequate sample size is essential to ensure any trial can provide reliable answers to its research questions, but information needed for that assurance is lacking during pandemic. For example, in a trial in hospitalized patients comparing agents with respect to time-to-invasive mechanical ventilation or death, the key determinant of power is the number of events but assessing patient numbers needed to accrue that number of events requires knowledge of the event rate. Likewise, when the outcome measure is the WHO 8-point ordinal scale analyzed using a proportional odds model (a generalization of logistic regression), sample size and power depend on the proportions of patients in each of the 8 categories at the end of the trial, together with an assumption about the odds ratio [6]. Lack of knowledge about these and other parameters makes the idea of a blinded sample-size reassessment attractive. The idea is to examine a subset of within-trial data combined over arms to reassess parameters and sample size. Blinded sample-size recalculation has a long history (e.g., for binary outcomes [7]; for continuous outcomes [8]) and is widely accepted because it is informative and preserves the advantages of maintaining the treatment blind.

In the final days of designing ACTIV-1, -2, and -3, results for studies of remdesivir were just emerging, allowing some, but not all to emulate their endpoint. However, the remdesivir study did provide benchmarks for hypothesizing treatment effects of anti-inflammatory therapies, monoclonal antibodies, and other investigative agents. As time went on, some master protocols relaxed power requirements and focused on agents hypothesized to have very large effects (ACTIV-5). Power for outpatient master protocols was more difficult to model, given uncertainty of infection and hospitalization rates early in the pandemic and their fluctuation later in the pandemic.

Given the paucity of preliminary outcome data, and in some cases lingering uncertainty regarding the statistical approach, several ACTIV programs provided for an interim assessment of statistical power and sample-size adequacy that also included futility-stopping boundaries. With the anticipated number of agents to be studied, it was deemed important early on to use aggressive futility boundaries for stopping agents unlikely to exhibit the moderate effect sizes targeted.

Some trials incorporated futility guidelines based on conditional power, the conditional probability of achieving a statistically significant benefit at the end of the trial, given current results and a projection for future data [9]. In one instance, consideration was given to stopping for futility if conditional power under the originally hypothesized treatment effect dropped below 20%. Other trials incorporated a more formal approach using beta spending functions that could range from quite conservative to quite aggressive [10]. The ACTIV-1 trial took this approach comparing immune modulators abatacept, infliximab, and cenicriviroc to placebo, and used a moderately aggressive beta spending function that tended to stop earlier than conditional power guidelines; whereas, ACTIV-6 included a screening endpoint to either fail or advance agents early based on the posterior probability of efficacy. Advancing agents were then monitored for efficacy on the primary endpoint, and for futility using the predicted probability of success.

### Flexible inclusion/exclusion (eligibility) criteria

While challenges with inclusion/exclusion criteria are not unique to master protocols, the desired efficiency gains in implementing a master protocol do require standard eligibility criteria that can be applied across all agents. Departures from standardized criteria should be minimized as they introduce complexity of implementation and loss of efficiency. To this end, like other COVID-19 platform trials [11] during the pandemic, the ACTIV master protocols inclusion/exclusion criteria targeted inclusiveness and generalizability within the parameters of each platform’s targeted setting (inpatient or outpatient), the candidate therapy class (e.g., immunomodulatory agents), and the targeted COVID manifestation (e.g., ARDS). All master protocols specified eligible patient ages (mostly ≥ 18 years), care venues (inpatient versus outpatient), and the required SARS-CoV-2 diagnostic testing (Table 2). With the exception of the three platforms on the master protocols studying thromboembolism prophylaxis that allowed enrollment of patients without COVID-19 symptoms, all master protocols also specified the COVID-19 manifestations required for trial eligibility using explicit but broad criteria unrestricted to historical norms. For example, because traditional definitions of ARDS were poorly suited for a respiratory viral pandemic featuring extensive use of non-invasive respiratory support strategies for patients with very severe hypoxemia [12,13], the ACTIV-3B platform investigating COVID-19 ARDS therapies included patients receiving high-flow nasal oxygen or non-invasive positive pressure ventilation. Other master protocol eligibility criteria were non-specific or implemented to simplify the trial; ability to consent and suitability for enrollment per investigator judgment were specified eligibility criteria in 9 of 11 master protocols, while ability to adhere to study procedures, lack of other prior or planned experimental therapy or trial, and absence of pregnancy (sometimes at the behest of regulatory authorities) were each specified in seven master protocols. Such broad criteria provide considerable latitude for a pandemic setting and maximize generalizability. Building on this experience, ACTIV has begun trying to implement lessons learned through the initiation of a new forward-looking master protocol that specifies the broadest possible trial population, Strategies and Treatments for Respiratory Infections and Viral Emergencies (STRIVE): consenting adults hospitalized with evidence of respiratory infection who are able to adhere to study procedures, are felt appropriate for the study by the investigator, and are neither moribund nor imminently discharging from the hospital (Table 2).

**Table 2.** Inclusion and exclusion criteria specified in Accelerating COVID-19 Treatment Interventions and Vaccines and Strategies and Treatments for Respiratory Infections and Viral Emergencies master protocols

Eligibility criteria	Number of ACTIV master protocols incorporating criterion	Criterion included in STRIVE master protocol
Patient care level at enrollment (inpatient vs. outpatient)	11 (100%)	X
Age	11 (100%)	X
SARS-CoV-2 diagnosis method an associated eligibility time window	11 (100%)*	N/A†
Qualifying signs/symptoms and associated eligibility time window^§^	8 (73%) *	X
Ability to adhere to study procedures	7 (64%)	X
Absence of life-limiting condition (varying definitions and windows)	6 (55%)	X
Expected disposition (e.g., length of stay)	5 (45%)	X
		
Exclusions		
Investigator judgement	9 (82%)	X
Concurrent experimental therapies and/or trial participation	7 (64%)	
Pregnancy	7 (64%)	
Allergy to study medication	6 (55%)	
Conflicting medications and treatments	6 (55%)	
Comorbid or concurrent conditions	6 (55%)	
Breastfeeding	3 (27%)	
Incarceration/prisoner	2 (18%)	

ACTIV = Accelerating COVID-19 Treatment Interventions and Vaccines; STRIVE = Strategies and Treatments for Respiratory Infections and Viral Emergencies.

* One master protocol did not include an eligibility window for diagnosis and two did not include a window for COVID-19 signs/symptoms

† STRIVE platform is designed to be inclusive of respiratory infections generally and not specific to SARS-CoV-2 infection.

§ ACTIV-4A, -4B, and -4C did not require symptomatic COVID-19 and were all focused on prophylaxis of COVID-related thromboembolism.

Some but not all platforms incorporated exclusions for concomitant medications or conditions for which the platforms’ trialed medication class was contraindicated (e.g., conditions with elevated bleeding risk in ACTIV-4B and ACTIV-4C). Such criteria required revisions when new drug classes were added or dropped from a platform, such as occurred in ACTIV-4HT and ACTIV-6; these were handled through agent-specific exclusions. When sharing placebo controls among study arms in a master protocol, it is important to apply such agent-specific exclusions to the whole control group.

### Outcomes measure and optimal endpoints

Many challenges existed when choosing endpoints in an evolving environment. As discussed in the ACTIV statistical lessons learned manuscript in this issue, the platforms each overcame the lack of information about the disease and the changing clinical milieu in different ways. Early designs focused on mortality, using the World Health Organization’s clinical status scale as a secondary measure. With the release of ACTT-1 and RECOVERY results, attention turned toward recovery from critical illness, i.e., time to discharge. Subsequently, time to recovery assessed using daily symptom diaries was adopted for outpatient studies. ACTIV-1, ACTIV-3, and ACTIV-6 used intermediate endpoints for rapid evaluation of agents, and ACTIV-5 used aggressive screening rules to drop agents early. Despite considerable inbuilt flexibility, each of the platforms needed to adapt the approach to endpoints midstream, unplanned changes to endpoints are traditionally inappropriate in regulatory intent trials due to the desire to ensure full data for statistical power, which can be a challenge if the endpoint varies too greatly. There remains considerable work to be done to assess the optimal primary and secondary outcomes in COVID trials, and the ACTIV platform data can be used to inform such research.

### Industry partnership

ACTIV was designed as a public-private partnership [14]. This included not only the original founding 20+ companies but also the industry partners that provided their agents for each ACTIV master protocol. A key lesson was that the early partners could help in the design of the protocols through statistical design and modeling, even if they did not have an agent to enter the trial. As companies agreed to partner with the trials, in addition to donating the agent and placebo for the trials, each company dedicated expertise and effort to writing the master protocols and their specific appendix.

To be efficient, master protocols need to be highly standardized across agents with any agent-specific details kept to a minimum. This requires consensus from all companies participating in a master protocol. For ACTIV, this meant all industry partners had to agree to streamlined inclusion and exclusion criteria, identical endpoints, statistical analysis plans, dataset formatting and delivery, and processes to streamline drug logistics. Many agreed to share or pool placebo participants, which introduced the complexity of how to avoid cross-pollinating data across investigational products when some molecules finished earlier than others and used a shared placebo. While giving up some control is necessary, regulatory efficiencies and consistencies are gained in a master protocol as the advice on the protocol is provided in one meeting with FDA versus multiple separate meetings. All of these decisions and consensus require partnership and transparency in decision-making. Though the industry standard is a stand-alone trial per product, ACTIV learned that having the industry partners engaged in developing the compromises is vital to improving operational and statistical efficiency.

For trial conduct, in the early days company partners also helped to find and acquire viable trial sites, personal protective equipment, and other scarce resources for the conduct of the trials. One key lesson is that early engagement from industry partners was critical to trial success.

### Definition and simplicity in trial governance structure

Several key considerations were at play in designing governance. Expertise in biology, manufacturing, safety monitoring, regulatory oversight, and interactions as well as familiarity and experience with the cultural differences among academics, community hospitals, life science companies, federal funding agencies, and regulatory agencies is unlikely to exist within a single committee, let alone an individual. It was thus critical the platform trials have governance structures that were nimble and able to coordinate and guide distributed expertise. The general approach within ACTIV was to have an overall steering committee for the platform trial, with co-chairs specific to the platform. Steering committees were constructed to be diverse at multiple levels, including assuring that complementary and broad-ranging expertise was present. Flexible and transient subcommittees and working groups as well as informal consultations were employed for particular problems (e.g., a regulatory nuance at a site outside the US or a complexity around local management of investigational product).

While each master protocol was specific to a target population and the general approach was designed to be appropriate to that target population, the protocols did not specify individual agents for investigation. Individual agent trials (generally presented as an appendix to the master protocol) were led by trial-specific co-chairs and protocol development committees. However, the ACTIV teams acknowledged existence of high-dimensional interdependencies that meant decisions had to be made in specific contexts and with associated compromises, which meant the individual agent leads needed to tap into committees assembled by ACTIV with appropriate and complementary expertise. This allowed key problems to be addressed by people knowledgeable and passionate about them in a way that quickly surfaced concerns and areas for improvement. To assist with navigating the interdependencies between trials, some of the master protocols relied on a joint Trial Oversight Committee to help counsel on agent after the ACTIV Agent Selection Committee had recommended them to the trial teams, common regulatory issues, and changing pandemic landscape challenges.

The role of NIH and Foundation for the National Institutes of Health (FNIH) were crucial to success of the ACTIV platform. FNIH provided an infrastructure for collaboration among industry representatives and academics (as well as overseeing objective agent selection and prioritization by the ACTIV Agent Selection Committee), while NIH served as sponsor and IND holder for many of the trials. By moving the sponsorship to NIH, NIH was able to harness expertise while assuring that the public-private partnership could move in a fair and objective way that was not bound to industry governance even with drug manufacturers as active partners in the process.

### Regulatory

ACTIV study teams benefited greatly from early involvement by regulatory agencies, specifically the FDA. FDA Statistical reviewers with expertise in infectious disease clinical trials participated in discussions about study design and interim analyses, providing advice on approaches that would be acceptable for EUA of ACTIV agents. Clinical reviewers shared their expert knowledge about the agents or class of agents being investigated and advised the ACTIV teams on various aspects of protocol design, always with an eye toward EUA submissions. FDA leadership (CDER and OND Directors) joined meetings at appropriate times, helping move the protocols along.

Once protocols were developed, ACTIV teams interacted with clinical and statistical reviewers from a variety of therapeutic areas in seeking regulatory approval. FDA engaged medical review divisions outside of the anti-viral division to manage the large number of submissions and urgency of responses in this effort, which enabled accelerated review turnaround times. However, some issues were encountered due to unfamiliarity of these divisions with pandemic-related trials. A weekly touch base between leadership of USG-sponsored emergency response master protocols and leadership from FDA review divisions enabled faster implementation of emergency research response trials.

It is important to note that all the ACTIV protocols were intended to be global, requiring interactions with regulatory agencies beyond US borders. Despite strong desires to collaborate across global regulatory agencies and using the same ICH standards, harmonized regulatory requirements were not achieved and every country required an individual review, significantly slowing global implementation. ACTIV protocols began with the FDA approval, which varied in terms of speed of approval from weeks to months. After receiving FDA agreement that a protocol was safe to proceed, regulatory approvals in other countries were sought. Efforts were made to meet with the EMA in advance to attempt to accelerate approvals. Despite EMA approvals each EU country had to provide approvals. In the case of ACTIV-3, sponsors varied by country and were facilitated by the University of Minnesota working with the International Coordination Centers. This sequential approval process limited the speed at which any global trial started and will continue to do so if not remedied. In fact, as a consequence of this slow sequential process by the time a sub-study of the protocol received regulatory and ethical approval in some countries, that sub-study had closed. After so much effort, paperwork, and time to review by so many persons, this was enormously frustrating. Global goals of 100 days to effective safe vaccines and therapeutics are aspirational and will be impossible under the current fragmented global regulatory system. To ensure an ability to execute a rapid research response, collaboration across global regulatory agencies is necessary to ensure rapid launch of global trials in the context of a pandemic.

ACTIV study teams relied on DSMBs for ongoing review of safety data and formal review of interim analysis results for futility or early efficacy. These interactions were successful in most cases, but there were exceptions. One exception was during an early review of safety data for one trial, the DSMB advised continuation and the FDA advised cessation based on the same data summaries. The NIH sponsor stopped the sub-study, but upon review of the complete data following early termination, there was no evidence of greater numbers of adverse events in the intervention arm compared to the control suggesting continuation of the sub-study might have been warranted. Given this was the last sub-study for the trial, relatively little overall disruption to the trial was caused, but there were significant time and effort expenditures during the deliberation and early closure of the sub-study.

### Assembling networks to support platform trial design and conduct

From March 2020 to March 2021, ACTIV assembled a Clinical Trial Capacity Working Group (CTCWG) to help evaluate clinical trial network readiness. The CTCWG was charged with developing an inventory of clinical trial networks, including trial sites drawn from networks assembled by both the NIH and CROs, who could serve as potential effective COVID-19 clinical trial implementers. The CTCWG focused on identifying data from different populations and disease stages; leveraging infrastructure and expertise from across NIH, non-NIH networks, and CROs; establishing a coordinated mechanism across networks to expedite trials; tracking incidence across sites; and projecting future capacity. This team evaluated 54 networks that included over 647 sites and surveyed 35 CROs/SMOs for their capabilities. The CTCWG identified 80+ novel and scalable enhancements/efficiencies for the conduct of therapeutic and vaccine protocols. Their five innovation playbooks can be found on the FNIH ACTIV: Clinical Trial Capacity Working Group web page [14]. For most of the ACTIV master protocols, this group helped to identify base networks suitable for the conduct of each trial and brought in the lead clinical investigators for those networks to help design the protocol their networks would conduct. The CTCWG also assisted in helping these base networks find additional networks and sites to partner with to expand their US and global footprint. The exceptions were that ACTIV-1 was designed by the Master Protocol Subgroup of the Therapeutics-Clinical Working Group for ACTIV and later taken up by the National Center for Translational Science clinical trial networks, plus some enhancement; and the National Heart, Lung, Blood, and Sleep Institute assembled the Collaborating Network of Networks for Evaluating COVID-19 and Therapeutic Strategies (CONNECTS) [15], whose investigators designed the suite of ACTIV-4 master protocols. Finally, as the pandemic continued, there was a need for identifying further clinical trial-ready sites, so the industry-driven TransCelerate Biopharma [16] effort was contacted, and the ACTIV industry partners assisted in searching the TransCelerate databases for sites to be approached to be added to the ACTIV-2 and ACTIV-3 networks. The work of the CTCWG and the CONNECTS team showed that it is invaluable to have well-cataloged clinical trial networks and sites that can be readily activated for clinical trial conduct in an emergency situation. This was not the state of affairs at the start of ACTIV.

### ACTIV trials quality (risk of bias)

The Cochrane Collaboration has developed a tool for assessing risk of bias in randomized trials [17]. During a post hoc analysis of the ACTIV master protocols to assess the quality of the trials, the following criteria were evaluated for each trial based on Cochrane Collaboration guideline: Method of randomization; Allocation concealment; Blinding (Subject, Investigator, and outcome assessor); Deviations from intended interventions; Incomplete outcome ascertainment; and other sources of potential bias. Subsequently, each domain of potential risk was evaluated with the Cochrane algorithm for each ACTIV trial, with an assignment of low, some concerns, or high risk. Based on this algorithm, all the ACTIV trials were designated as low risk. In fact, an important feature of most ACTIV master protocols was the use of matching placebo to enable double-masking of treatment assignments, thereby eliminating a key source of bias often found in randomized, open-label studies, where this is an element of subjectivity in assessing the outcome measures.

## Conclusion

The approach that ACTIV took to address the COVID-19 pandemic provided numerous learnings for master protocol design both during a health emergency and during normal clinical trial practice. These lessons, summarized in Figure 2, as well as lessons previously reported [5], can be parlayed into future master protocols, as well as pandemic preparedness efforts. These lessons are already being applied to the STRIVE study, and to the master protocols for studying long COVID as part of the RECOVER initiative at NIH. These immediate applications of the ACTIV master protocol lessons learned and ability to test further iterations on design will help to position the field for a faster more cohesive response to the next pandemic.
